# Transferring research from a university to the United Kingdom National Health Service: the implications for impact

**DOI:** 10.1186/s12961-017-0219-3

**Published:** 2017-06-17

**Authors:** Helen Payne

**Affiliations:** University of Hertfordshire, School of Education, De Havilland Campus, Hatfield Business Park, Hatfield, Herts AL10 0NZ United Kingdom

**Keywords:** Knowledge transfer, University spin-off, Reflexivity, Research impact, Engagement, Academic entrepreneurship, Commercialisation

## Abstract

The aim of this article is to inform readers of the author’s reflections on the experience of transferring university-based research into the commercial sector, and of the processes and strategies employed when preparing for impact in so doing. Concepts for the transfer are illustrated by the author’s reflection on aspects that arose during the birthing and subsequent start-up of a university spin-off, Pathways2Wellbeing, a form of reflection-on-action. This is the vehicle for the adaption required to transfer research into the delivery of a specialised clinic in the United Kingdom National Health Service for people with medically unexplained, persistent, bodily symptoms such as fibromyalgia, chronic fatigue and chronic pain. It is hoped that the article will provide readers with an insight into how knowledge transfer can take place through engagement with stakeholders to create an exchange of knowledges to result in impact on health service policy for service users, despite the challenges, and the enablers that facilitated this process. The reflections on the process of knowledge transfer and the implications for impact are underpinned by relevant theory.

## Background

Researchers in universities, as major contributors in the production and distribution of research, have to be effective not only in knowledge dissemination but also knowledge transfer. Knowledge dissemination typically refers to activities undertaken by most researchers towards their peers (e.g. conference presentations and publications in, increasingly, open access, peer-reviewed journals) and tailored messages to a specific audience (e.g. briefings to stakeholders, educational sessions with patients, practitioners, and/or policymakers, media engagement). Knowledge transfer is more than the distribution of research outcomes and dependence on academic publication as a major tool for disseminating results. It denotes a collaborative and engaged process between the research and systems of care (i.e. teams, populations, policymakers and consumers) [1]. For the effective transitioning of academic research, others, especially outside academia, need to see the usefulness of the research to them for it to subsequently create an impact (make a difference).

What stops researchers from getting their research to those in healthcare policy who might be able to use it? Perhaps there are too many challenges and, together with a paucity of evaluation strategies, researchers may not feel equipped to make the cross-over from academia to a real-world context. A review of studies evaluating knowledge transfer by Mitten et al. [2] reports on the inadequate evidence base for evaluating knowledge transfer, its challenges and limitations, and calls for a rigorous evaluation of strategies. A recent review by Elueze [3] proposes the notion of intermediaries or bridges to support researchers in healthcare as ‘knowledge brokers’ to develop networks and relationships with, among and between producers and users of knowledge. By providing linkages, sources of knowledge, research evidence and marketing insights they can link researchers to users of research evidence enabling collaboration to identify issues, solve problems and promote evidence-informed decision-making in policy and practice.

Strategies referring to the importance of interaction involving the interchange of knowledge between, in this case, the United Kingdom National Health Service (NHS) Clinical Commissioning Groups (CCGs, the policymakers/funders), patients and doctors (research-users) and the researcher-producer are crucial. From the involvement of the author as researcher in this particular knowledge transfer, via a university spin-off, it became clear she needed to considerably adapt the knowledge produced by the research for it to be embraced by others in the NHS. Adjusting the research and engaging with the relevant stakeholders who could make use of it, made it accessible to them, and thus enabled it to become adopted by them, which in turn created reach, significance and impact.

Commercialisation, defined as intellectual property creation and academic entrepreneurship, is quite different from traditional academic engagement practices such as collaborative/contract research, consulting and other knowledge exchange projects [4–7]. In the example below, the transfer of the research knowledge was formulated by the creation of a commercial clinical service designed for groups of people with medically unexplained symptoms (MUS) and employing a methodology specifically planned for their treatment in the NHS primary care community in order to help them self-manage to live well with their chronic symptoms.

## Knowledge transfer

Knowledge transfer is a term used to encompass a broad range of activities to support mutually beneficial collaborations between universities, businesses and the public sector. It works best when people meet to exchange ideas and involves dissemination, awareness- raising, engagement and impact. It refers to the challenge of transferring knowledge from one organisation to another and seeks to shape, adapt and distribute knowledge to ensure its accessibility for other users. It is a continuum of processes and activities that brings researchers, decision-makers and end-users together. One definition, from Owen [8], is as follows:

“*Knowledge transfer is about transferring good ideas, research results and skills from universities and other research organisations, to business and the wider community to enable innovative new products and services to be developed … evolved to often include the exchange of information through networks and takes place when existing information is recombined in a new way. The Government’s aim is to promote the transfer of knowledge generated and held in higher education institutions and public sector research establishments to the wider economy to enhance economic growth*.”

To know the societal impact takes years and it is difficult to identify causality between a study outcome and impact since the pathways are often diffuse and unclear. On the other hand, in some cases, especially where there has been a collaborative approach, it can arise quite rapidly and directly. Yet, impact seems to need consideration from the outset of the research trajectory. Crafting case studies to illustrate impact, as is the case in the Research Excellence Framework (REF), although laborious, appears to be “*the best way of measuring the complex phenomenon that is societal impact*” [9]. The Stern review [10] recommends that, for the forthcoming REF, “[impact case studies], *need not solely focus on socio-economic impacts but should also include impact on government policy, on public engagement and understanding, on cultural life, on academic impacts outside the field, and impacts on teaching*”. However, The Higher Education Funding Council for England point out in their ‘Consultation on the Second REF’, that “*The broadening and deepening* [recommended by Stern] *included some areas that fell within the definition of impact for REF 2014…*” ([11], paragraph 78).

The example herewith can demonstrate impact on public engagement, government policy as manifested in NHS localities, and on teaching (e.g. doctors and doctoral programmes in clinical psychology). The Stern review also states that “*in order to encourage long-term, interdisciplinary research endeavours, we recommend that ground breaking academic impacts such as research leading to the creation of new disciplines should be included*” [10]. In this case example, the interdisciplinary nature of the research (integrating health psychology with embodied approaches to change and transformation – dance movement psychotherapy) has been the basis of a new discipline manifested by this tailor-made clinical service employing an embodied approach.

Knowledge transfer as identified in a report by van Vught and Ziegele [12] is, according to Bormann [9], “*concerned with assessment of: (a) social, (b) cultural, (c) environmental, and (d) economic returns (impact and effects) from results (research output) or products (research outcome)*”; the term ‘products’ could be replaced by ‘service’ in this case.

There has been a narrowing of the previous wide gap between knowledge created from research in universities [13] and knowledge used [14] in, for example, business, policy and wider society with reference to impact and effects. Perkman et al. [15] reviewed the literature on university–industry relations to find major developments in the commercialisation of academic research. Martinelli et al. [16] give an excellent illustration of the process of becoming an entrepreneurial university, in which a considerable number of researchers engage in knowledge exchange processes with industry and other non-academic partners, pointing to knowledge exchange relationships and faculty attitudes as crucial elements. In contrast to these studies, a Canadian study of knowledge transfer in universities by Landry et al. [17] found that researchers transferred knowledge more actively when there was no commercialisation or protected intellectual property, and that only some researchers in some fields were active in knowledge transfer. They found that a focus on users’ needs and linkages between researchers and research users were the only two common determinants explaining knowledge transfer, a finding which this author can support from experience. Other determinants influencing knowledge transfer varied from one research field to another, suggesting different policies would be required to increase knowledge transfer in different research fields.

In the United Kingdom health sector, there has been a determined effort to transfer knowledge from studies conducted in hospitals into optimal patient benefits [18], but none to the author’s knowledge referring to health services developed outside the NHS itself. Data from 36 studies of the impact of multi-project programmes of funded health research based in the NHS shows that many projects in some programmes, particularly those which were needs-led or collaborative, report making an impact on policy 35% (range 5–100%), practice 32% (10–69%), combined category 64% (60–67%), and health gain/health services 27% (6–48%) [19]. Unfortunately, however, a study in general healthcare by Straus et al. [20] showed that decision-making groups within the public, patient, healthcare professionals, managers and policymakers all fail to use research evidence to inform their decision-making. Furthermore, according to a systemic review by La Rocca et al. [21], the effectiveness of strategies for the transfer of knowledge from research into public health has been lacking. They also found that conclusions about interventions should not be taken in isolation from consideration of the characteristics of (1) the knowledge that was being transferred, (2) providers, (3) participants and (4) organisations. For university-based research to be useful outside a university setting it has to be accessible to the non-specialist as well as the specialist (a process of translation). Strategies for transfer, La Rocca et al. [21] claim, need to incorporate the characteristics of the host organisation/end-user as well as those of the knowledge to be transferred. In other words, the knowledge needs to be adapted for the context in which the product or service is to be used and a knowledge ‘exchange’ set up. Instead of the term knowledge ‘transfer’ the term knowledge ‘adaptation’ might be more suitable. In the case example presented herewith, it was indeed important to integrate the characteristics (including priorities and/or needs) of the NHS CCGs, the doctors and the patients (together with those of the University) with the research knowledge. This synthesis of knowledges changes the knowledge to suit the context. Bowen and Graham [22] summarise what is known about what works in the American health sector to promote evidence-informed action/utilisation, tracking from a linear focus on research transfer to intricate strategies for user-engagement, challenging the evidence and assumptions on which current knowledge-to-action activities are based.

## The spin-off

Spin-offs are found in United Kingdom higher education institutions (HEIs) as part of a planned methodology to promote knowledge exchange/transfer and to maximise impact from research. From data collected by The Higher Education Business and Community Interaction Survey for the academic year 2013–2014 [23], there was a continuing increase in the exchange of knowledge between HEIs and the public, private and third sectors in the United Kingdom, although the number of spin-offs decreased slightly. In 2012–2013 (the year the spin-off called Pathways2Wellbeing was formed) there were 126 HEI sponsored spin-offs created with some HEI ownership. In that year, the total number of spin-offs with some HEI ownership that had survived for more than 3 years was 806 out of a total of 1069 active firms [23]. Consequently, there is a small drop-out of spin-offs both before and after the 3 year watershed. However, according to that same report there have been increasingly more incentives than barriers for staff to engage with business and the community since 2000–2001 (at 57% of HEIs). However, in a recent United Kingdom example [24], there is a cautious note that the changing professional identity of the researcher can lead to the development of a hybrid role on the edges of the HEI, which, is argued, might become untenable as time goes on. The author can concur that this hybrid role can be experienced in both the university and NHS setting. With a foot in each setting it can be a complex process differentiating between the researcher role in the University and the practitioner and business roles in the spin-off.

The term adaptation in the context of knowledge transfer will usually involve knowledge that requires replication and knowledge that requires adaptation [25]. The Canadian Institute for Health Research [26] suggests that knowledge translation is a broad concept involving dissemination, transfer and assessment of technology, ethical considerations, knowledge management and utilisation, exchange between researchers and users, implementation research, synthesis of research results in the global context, and development of consensus guidelines.

The vehicle for the knowledge ‘adaptation’ in this case example was through the creation of a university funded spin-off called Pathways2Wellbeing (P2W) as a method to engage with the NHS to fund the delivery of a research-informed service. The author’s University decided to create, and invest in, this organisation, which had as its objective the delivery of a specific, tailor-made clinical service in the NHS. P2W is therefore the vehicle for the dissemination of the research through a service based on the research [27–30]. The University retains a proportion of stock in the company to sell off as shares in due course and monitors it through financial and activity reports four times a year.

The intervention at the heart of this clinical service is designed to support people with MUS in primary care. They are a large but marginalised patient cohort with persistent symptoms such as fibromyalgia, irritable bowel syndrome, chronic fatigue, chronic pain and others for which tests and scans, etc. always come back negative. Many have depression and/or anxiety, are without a cohesive patient voice, with complex and long-term needs, and for whom there has been no alternative solution. These are very high health-utilising patients, frequently visiting their doctor (more than five times per year) and for longer than the allocated 10 minutes, with numerous visits to hospital/accident and emergency services and in receipt of much medication. These conditions cost £18 billion in 2008 according to Bermingham et al. [31], and £3.1 billion of this to the NHS, which is approximately 10% of the NHS budget.

There is research evidence [32] that this large patient group are mostly unwilling to attend mental health services due to their explanatory model of their bodily symptoms being physical, and the stigma attached to mental health and wellbeing services. From the many patients referred to P2W, less than 10% withdraw once they have begun the course. The patient experience surveys report ‘good’ to ‘very good’ overall satisfaction levels with the service [33]. Therefore, it follows that the P2W MUS clinic appears to be more acceptable than referrals to the wellbeing/mental health psychological services for this cohort of patients. In line with embodied social cognition theories, the intervention, called The BodyMind Approach™, highlights the central role of the physical body in influencing the mind, emphasising the link between altered (physical) interoceptive subjective experience, disturbed inter-subjectivity and neurobiological dysfunctions. In this view, “*sensorimotor capacities are themselves embedded in a more encompassing biological, psychological and cultural context*” ([34], p. 71). Thus, this service aims to increase self-regulation through ‘lived body’ experiences, such as body awareness practices, in order to support patients to self-manage. This acts as a protective factor since it reduces the degree of symptom distress, anxiety and depression, sustained over the longer term, to prevent any worsening and the resultant higher costs to the NHS.

## The engagement process

Without engagement, knowledge transfer and potential impact cannot take place. With reference to engagement, Harris [35] notes that:

“*…engagement denotes the need for* **closer relationships between researchers and research users** *, requiring co-creation of content and greater involvement in the promotion of results. Achieving this is only possible through active participation in networks, through which research findings and concepts circulate and are gradually filtered. Think tanks, advocacy coalitions, policy streams, policy communities and national and regional networks are frequently cited as being important in this regard*.”

Patient and public involvement in, and understanding of, research has been a growing priority for funders and is referred to as important in the Stern report [10]. Subsequent to the initial research, a network of patients have been invited to become engaged as P2W ambassadors, in commenting on further research designs/bids and in evaluations of the service in order to inform subsequent delivery. Furthermore, the website, brochures and other marketing resources have been commented upon by patients, self-help patient organisations and doctors. Output has been communicated to healthcare professionals and the general public by the team and patients. Patient networks, nationally and regionally, have been supportive in advertising courses, publishing articles and blogging about the benefits to patients.

In order to engage and inform the wider local community generally and raise awareness with policymakers a website has been developed (www.pathways2wellbeing.com). This attempts to engage with potential trainees in the facilitation of the group intervention, commissioners, doctors, patients and the public through blogs, courses and other resources such as a doctor referral system. Twitter [36] and YouTube [37] have been helpful in spreading the word. Inquiries from patients, doctors and those interested in training as group facilitators for the service are received as a result of this exposure.

A wide range of strategies have been employed to engage with patients, doctors and commissioners, including:

- Communicating with patient groups and networks
- Networking with individual doctors and practices and delivering talks on training courses for doctors
- Delivering talks on continuing professional development courses to mental health professionals
- Training mental health professionals in the intervention as group facilitators
- Presenting to consultants in neurology, gastroenterology, rheumatology, etc.
- Delivering free public talks in local community centres and universities
- Writing for popular medical magazines [38], conducting local/regional radio interviews
- Presenting at the various policymaker/commissioner events and networking at health conferences [39]
- Giving talks/working closely with the voluntary sector

To ensure that these forms of engagement are effective, that the message is perceived as useful knowledge, and that it has an influence, many relationships were built. Although essential to successful adoption and impact in this illustration, these took a long time and were hampered by NHS staff frequently changing roles or leaving, which added to the frustration and delay in implementing the knowledge transfer. These relationships, for example, with key commissioners, locality managers, doctor surgery practice managers, the voluntary sector, patient networks/self-help groups, doctor training organisations and the mental health training sector, were crucial and needed to be two-way, long-term and trusting.

Together with these methods of engagement, P2W developed a strategy to identify what was required and put into place methods of attaining these targets. That is, it designed the knowledge exchange (i.e. methods for raising awareness through engagement as above) but also had to learn to be flexible enough to adapt to the changing environment of the NHS. At the time of inception, the NHS changed from Primary Care Trusts to CCGs and the Strategic Health Authorities were disbanded. This was an immense NHS re-organisation, medical staff had many priorities to juggle. P2W needed to acknowledge this and adapt its unique selling points to these new areas of focus. Fortuitously, a champion or ‘knowledge broker’ was identified in one CCG who remained in post long enough to support the first pilot service deliveries.

Simply informing people of the research and its meaning/benefits was insufficient for stakeholders to commit to piloting a novel intervention though. The P2W service had to be adapted, piloted in their locality and then adapted again. Furthermore, output needed to mirror the previous positive results for patients and the NHS from the research for commissioners (who are now doctors since the implementation of CCGs) to be convinced of effectiveness.

The research end-user knowledge needs and priorities have to be recognised and any communication has to be with these in mind. For the first pilot, ex-patients and the CCG commissioner-champion (in the role of knowledge broker) were able to support P2W with sharing these needs and priorities. For example, for patients, it was the decrease in levels of symptom distress that they felt should be the highest priority when considering output from the intervention. For commissioners, it was the reduction in costs in primary and secondary care, the need to manage an annual 3% increase in healthcare demand and, consequently, the need for an increase in doctor capacity due to a lack of sufficient numbers (since recruitment was, and is, low together with high numbers retiring/leaving the NHS contrasting with the increase in population). Service delivery outputs were therefore presented with these priorities/needs articulated. The knowledge broker understood the various perspectives of their stakeholder group, the CCG. Engaging with this local knowledge broker was crucial to making links and brokering the first pilot and the later commissions.

In this example, there were three communities that P2W had to involve, i.e. the CCG, the patients and the doctors. P2W acted as the interface between these three to embark on a communication process to discover each of their needs and priorities in order to translate and adapt the research into a service that responded directly to each. P2W became the connecting conduit between these three stakeholders and the University and thus became, not only the knowledge disseminator, but also the knowledge receiver (via feedback from commissioners, doctors and patients) – an exchange of knowledges.

In a study by Poliakoff and Webb [40], four factors were identified that predicted scientists’ intentions to participate in public engagement activities, over and above their past actions, namely (1) attitude (whether participation was regarded as positive), (2) perceived behavioural control (beliefs about whether participation was under their control and that they were capable of public engagement activities), (3) past behaviour (the extent of previous participation in public engagement activities), and (4) descriptive norms (whether scientists believe their colleagues participate). With reference to these factors, the author can echo that they were indeed present. Furthermore, Poliakoff and Webb [40] suggest educating scientists about the benefits of public engagement, offering skill-based training to foster perceived behavioural control and messages to increase awareness of colleagues’ participation in public engagement activities. In keeping with these, the University, which conceives of itself as business-facing and entrepreneurial, designed a planned strategy for encouraging public engagement in research activities. For example, it offers researchers courses in how to work with the media, presentation skills, the importance of impact, developing negotiation skills, writing for public consumption etc., public engagement activities and skills development.

Even with such support some researchers still need to be courageous to take that first step into the public domain. Universities can help them by providing a ‘knowledge broker’, taking heed of the four factors above and by instilling a positive attitude about sharing research, supporting researchers to feel that they have the skills and capability to reach out into the wider world, and by demonstrating that colleagues participate in public activities. These initiatives could help to further promote the development of impact from research in HEIs. Still, raising awareness/public engagement in preparing for impact is only a first step towards impact, it is not impact in and of itself.

## Impact

Impact is achieved from research when the knowledge base is built upon and there are benefits to health, culture, economics or policy. The elements of ‘reach’, ‘significance’ and ‘impact’ in the previous, and forthcoming, REF (the system for assessing the quality of research in United Kingdom HEIs) resulted in the need to go much further than communicating research findings in academic peer-reviewed journals or at conferences. The drive now is to facilitate exchanges in knowledge and understanding to those outside academia and to establish and evaluate impact. Hence, researchers, from the outset, need to plan for the promotion of engagement and consider strategies for achieving and evaluating the societal effects from the research, which, in this case example, are the influences on health professionals, commissioners/policymakers, patients and other stakeholders in the NHS such as those involved in public health. For example, the number of referrals to the clinic from General Practitioners and other health professionals, the number of commissions, changes in policy as a result of the innovation, the effectiveness on patient symptoms, acceptability, engagement and commitment to the intervention, and calculating any cost savings to the NHS.

The previously noted forms of engagement, subsequent to awareness raising, require a pre-planned evaluation as to the use to which the research has been put by the stakeholders to assess levels of impact. The evidence derived from collecting data on usage/influence (bibliometrics or otherwise) will then form the basis for concluding the degree of reach, significance and impact. Methods for evaluating whether (and how) the research has challenged assumptions, changed views, influenced policy and/or benefitted society need careful thought.

Researchers generate new knowledge; however, understanding its relevance and significance in the wider world is a further step to be taken if it is going to result in changing any aspect of public policy and/or society. The inspiration for the research was, firstly, the large number of primary care patients with MUS (previously termed psychosomatic conditions) and the huge gap in NHS services for them. Secondly, consideration of how the author’s background in embodied psychotherapy (integrating psyche and soma) might be applied to this population and a recognition of practice-based evidence from her private practice indicating that it might be effective. Thirdly, the high costs to the NHS for this patient population. Possible contributions to impact were thus identified at the outset. Subsequently, the research study was generated. Furthermore, a study outcome was the identification of what was important to patients in terms of benefits (i.e. reduction in symptom distress and feeling able to self-care going forward).

However, identification of what was important to commissioners and doctors was far more demanding when the CCGs were new, had so many other priorities and enormous organisational changes to deliver, at a time of austerity and a shortage of health professionals. Ascertaining their priorities and then adapting the communication to them about why these research findings could be significant has been key in the author’s experience. This meant re-framing the patient and NHS benefits in a way that made sense to them by, for example, for the CCGs, calculating the costs saved, wastage reductions and reliable change in terms of patient benefits as a result of the clinical intervention delivery. Consequently, learning about the stakeholders’ priorities and needs is important when researchers are making a start on the adaptation process.

For impact to be shown in this example there needed to be evidence of a positive change in NHS policy and/or best practice for supporting people with MUS as a result of the knowledge transfer to achieve impact. The commissions gained demonstrate this impact on NHS policy and service development. Furthermore, best practice recommendations and those reliable changes [33] measured as benefits for patients indicates impact since patient self-management and overall wellbeing both improved after the treatment and 6 months later when compared with pre-treatment assessments. Practice-based evidence is collected and analysed from patient assessments and feedback which, to date, has mirrored the original research results in patient benefits and savings to the NHS [41, 42]. Furthermore, cost savings to the NHS have been calculated to show that it was an ‘invest to save’ initiative. This provides supplementary evidence of impact. It took over 4 years for any impact to emerge.

The REF is likely to continue using case studies as the main method to measure impact [43]. Assessment of impact can inform funding bodies and research institutions in their strategic planning [44]. However, assessing impact is a complex process as there are many ways of generating and using knowledge. A recent review by Greenhalgh et al. [45] of six established approaches to assessment of impact, namely Payback, Research Impact Framework, Canadian Academy of Health Sciences, monetisation, societal impact assessment, and the United Kingdom REF, including metrics and electronic databases concluded that (1) one size does not fit all; (2) robust and sophisticated approaches are labour-intensive and not always feasible or affordable; (3) whilst metrics may capture direct and proximate impacts, more indirect elements of the research–impact link can and should be measured; and (4) research on research impact is rapidly developing with the prospect of new and automated methodologies. It is interesting to note that the impact of the American National Institute of Health's Mind-Body Interactions and Health [46] was assessed using the Payback Framework, which was developed in the United Kingdom for the Department of Health’s Research and Development Division.

Another recent review of assessment of impact from the funder’s perspective (the Health Technology Assessment programme of the National Institute for Health Research) [43] found that the Payback Framework with adaptations remained the most common approach. It recommended a consideration of how case studies may be improved for a more systematic assessment. Furthermore, they conclude that case studies tend to be biased towards high-impact rather than low-impact stories, although experience from other industries indicates that much can be learnt from the latter. Although some United Kingdom research funders are adopting Researchfish® to capture indexed research publications, it has yet to be established whether it can collect non-indexed outputs and activities [43].

### The challenges

The main objective for P2W has been to gain commissions in the NHS in order to demonstrate impact; consequently, all efforts have been devoted to activities associated with that objective. Despite the University’s support for P2W as a vessel for the spin-off, the transition from university-based research studies to reach the patients who could benefit from the research has been a huge leap.

The time it takes for the researcher to adopt a commercial relationship with the research and the market place, and to translate research into services, taking account its characteristics and the features of the host and end-user, can be extremely complex and drawn-out. Furthermore, there needs to be time allocated to prove that the research results are mirrored in the marketplace. Then, even more time is required for the spin-off to generate any significant financial returns. It is now acknowledged that the timescale taken for the commercialisation of intellectual property linked to medicine, being so complex, is especially long due to, in this case example, the need for relationship building with a range of key stakeholders, finding and then making contact with the right person to speak to, understanding the market and commissioning process and the needs of each ‘audience’, the complexity of the health service, and General Practitioner practice accessibility. When compared to the application of drugs or surgical interventions, the design and development of operationalising and evaluating a highly complex behaviour change service, when developed outside the NHS, is even more drawn out. Trials cannot provide knowledge about why an intervention was successful or not, or if the theory and evidence was appropriate. An integrated quantitative and qualitative pilot study allows for sound judgment of the transferability of potentially effective services.

It was soon discovered that gaining commissions in the NHS is enormously complex for a clinical service innovation developed outside the NHS even though there was evidence that this one solved a range of problems. The team had to gain considerable knowledge and understanding of the health service and how it was articulated locally.

Research in the transfer of knowledge, such as that by Grimshaw et al. [18], suggests that planned knowledge transfer for healthcare professionals and consumers is more likely to be successful if the strategy is informed by an assessment of likely challenges and enablers. La Rocca et al. [21] conclude from their study that no singular knowledge transfer strategy was effective in all contexts. Since knowledge transfer is context specific, it is proposed that a suitably informed ‘knowledge broker’, ‘translator’ [47] or, as it is termed in education, ‘linkage agent’ [48], could offer vital support to adapt the research knowledge produced to make it easier to adopt and use by others. Furthermore, their experience in healthcare would be critical for considering challenges and enablers, assessing them and providing an understanding of the context for a successful transfer. P2W was fortunate to have identified an NHS commissioner-champion, who acted as the knowledge broker, to fulfil some of these important aspects of knowledge transfer and exchange.

With only three in the P2W team, resources are naturally limited and this is challenging given the enormity of the task. Moreover, those involved have only a small amount of time due to other commitments and/or full time jobs. Therefore, the project has been organised on a very part-time basis and, for the author, in addition to university duties, voluntary and in her own time. Since the budget was limited, much personal time and energy has been necessary. Even when there was a full-time business manager, the time and effort required to manage and support the manager was excessively heavy. Consequently, there have been a number of time-resourced activities which could not take place, for example, designing a marketing plan.

Sometimes, it has felt like there were just too many challenges to overcome given the few resources. The enthusiasm for the service maintained momentum in the short term. Yet, motivation needed to be sustained in the longer term too, so this became a test. Seeing the service making such a profound difference to people’s lives over time has gone a long way to facilitating the necessary continuous commitment from the team and associates (for example, group facilitators).

Furthermore, ownership has been developed by, for example, one of the team sharing in the authorship of academic publications. On-going commitment to P2W had also been sustained by buy-out time from some university duties for the author for a short time, and by providing small financial rewards for others. Such strategies to gain the ‘buy in’ from the key personnel involved is essential to maintaining long-term commitment in a spin-off.

The availability of positive research results does not assure its adoption and utilisation by potential end-users. The way the research results are presented can be either an incentive or a barrier to the adoption of knowledge in a community of practice [49]. For example, to commissioners, results were presented as cost savings per patient as well as clinical benefits to patients. It was important to communicate to doctors that not only does the service give patient benefits but also increases doctors’ availability to see other patients to whom they could offer help (especially important at a time of doctor recruitment shortages). To patients, the nature of the service and the expected benefits (such as self-care, reductions in the need for health appointments, symptom distress, anxiety and/or depression and increases in activity and wellbeing) that can be anticipated for them as individuals were outlined.

P2W had to make the research comprehensible and accessible to doctors to nurture their interest in the benefits for them (and their patients) to persuade them to become early adopters of the service and make referrals. However, making contact with them was difficult (perhaps due to their excessive work-load and the bureaucratic expectations) such as making appointments with surgeries to explain the service and referral procedure to doctors. Although the knowledge broker had introduced the service and promoted it via a doctor-champion at various doctors’ meetings, following this up by engaging directly with doctor surgeries was extremely problematic. Even obtaining the practice managers’ contact details was a major hurdle.

Unfortunately, in NHS primary care, the local features, traditions, norms, changes in structure, staff leaving/absence, priorities, government cuts and past experiences were barriers to gaining commissions. Every doctor’s surgery is different with varied funding arrangements (surgeries, as independent contractors to NHS England, receive a global sum and payments for quality and outcomes, enhanced services, seniority, premises, dispensing services, information technology, etc.), which complicates any business case and communication strategies. P2W had to spend a great deal of time assessing the context and features of a potential delivery, those individuals to target and the methods of approach to take whilst at the same time keeping the goal in sight, i.e. gaining a commission.

### Enablers

In contrast to the challenges, there were a number of enablers present in the NHS at the time of P2W’s inception. For example, the Five Year Forward View from 2016/2017 [50] aims to seriously encourage preventative factors to reduce later dependency on the NHS, to shift investment from acute to primary and community services, and to cut health-related unemployment. The government requirement to increase the availability of family doctors and reduce appointment waiting times, and the initiative to increase patient choice, especially in the psychological therapies, began. The P2W service offered a reduction in the number of visits a patient with MUS made to their surgery, thus reducing waiting times for appointments, increasing doctors’ availability to see other patients at a time when doctor numbers are falling significantly in the United Kingdom. Since most MUS patients are unaccepting of mental health interventions the P2W service presented a choice. A Department of Health policy to integrate physical and mental health for long-term conditions (of which MUS is one) was especially suited to this new service since it aims to integrate body and mind for people with MUS. The drive to reduce wastage, seek quality in services and improve value for money by CCGs supported the way P2W presented its business case. Moreover, the ambition across the whole of the NHS towards self-management, particularly for long-term conditions, has facilitated the case for this clinical intervention, which supports patients to do just that post intervention. This results in fewer doctor, accident and emergency and hospital visits, the reduction of which is a Department of Health target, together with goals for the reduction of costs to the health service and society. These drivers have been facilitators in the design of the P2W business case put forward to CCGs.

## Conclusion

P2W, together with patients and commissioners, has reflected on the knowledge transfer process over time in order to learn and refine its service delivery. This helps in cultivating the sustainability of the delivery and in transferring new knowledge to other commissions in due course. Each step in the process needs iterations; therefore, feedback loops are necessary. It is recommended that an evaluation strategy of knowledge transfer activities includes documentation of such feedback loops (resulting in the co-production of the knowledges) indicating the complexity of the system with which the transfer has been conducted. Furthermore, the way the exchange has been conducted with reference to different audiences/stakeholders, the challenges and how they were overcome, and the enablers and how they were employed to achieve impact might be included in any assessment of impact. For health initiatives, such as the example herewith, the duration allocated for impact would need to be at least 5 years, according to the author’s experience. Health research funders will continue to evaluate impact from studies they fund. It has been recommended that they review the contribution of case studies and expand on linking trials to meta-analyses and to guidelines [43].

The limitations and challenges to securing impact, especially for a service in the NHS, require substantial resources (including a knowledge broker) to be made available to any start-up university spin-off to help identify and resolve such limits if the transfer of knowledge is to be successful. Lawson and Potter [51] demonstrated determinants of knowledge transfer, within inter-firm new product development projects, as the buyer’s learning intention, supplier’s response, and the characteristics of the relationship and knowledge to be transferred. Individuals and organisations involved in knowledge transfer may need to consider processes on many levels, including social and contextual aspects, as pointed out in the literature review on knowledge translation by Oborn et al. [52].

Although P2W has had set-backs to this knowledge transfer process, and despite the immense tests, with the persistence of the team and some enablers in the NHS it is nevertheless beginning to achieve impact. For example, there has been another trial in one CCG subsequently followed by a commission in two further CCGs together with the author being co-opted onto the NHS England Task Force for medically unexplained symptoms.

## Abbreviations

CCG: Clinical Commissioning Group
HEI: higher education institute
MUS: medically unexplained symptoms
NHS: National Health Service
P2W: Pathways2Wellbeing
REF: Research Excellence Framework
